# Mechanical Properties of Two-Dimensional Metal Nitrides: Numerical Simulation Study

**DOI:** 10.3390/nano14211736

**Published:** 2024-10-29

**Authors:** Nataliya A. Sakharova, André F. G. Pereira, Jorge M. Antunes

**Affiliations:** 1 Centre for Mechanical Engineering, Materials and Processes (CEMMPRE)—Advanced Production and Intelligent Systems, Associated Laboratory (ARISE), Department of Mechanical Engineering, University of Coimbra, Rua Luís Reis Santos, Pinhal de Marrocos, 3030-788 Coimbra, Portugal; andre.pereira@uc.pt (A.F.G.P.); jorge.antunes@dem.uc.pt (J.M.A.); 2 Abrantes High School of Technology, Polytechnic Institute of Tomar, Quinta do Contador, Estrada da Serra, 2300-313 Tomar, Portugal

**Keywords:** 13th group atoms, metal nitrides, nanosheets, elastic constants, finite element model, numerical simulation

## Abstract

It is expected that two-dimensional (2D) metal nitrides (MNs) consisting of the 13th group elements of the periodic table and nitrogen, namely aluminium nitride (AlN), gallium nitride (GaN), indium nitride (InN) and thallium nitride (TlN), have enhanced physical and mechanical properties due to the honeycomb, graphene-like atomic arrangement characteristic of these compounds. The basis for the correct design and improved performance of nanodevices and complex structures based on 2D MNs from the 13th group is an understanding of the mechanical response of their components. In this context, a comparative study to determine the elastic properties of metal nitride nanosheets was carried out making use of the nanoscale continuum modelling (or molecular structural mechanics) method. The differences in the elastic properties (surface shear and Young’s moduli and Poisson’s ratio) found for the 2D 13th group MNs are attributed to the bond length of the respective hexagonal lattice of their diatomic nanostructure. The outcomes obtained contribute to a benchmark in the evaluation of the mechanical properties of AlN, GaN, InN and TlN monolayers using analytical and numerical approaches.

## 1. Introduction

Two-dimensional (2D) metal nitrides (MNs) are attractive emergent materials with important forthcoming applications in advanced electronics, the light industry, energy storage and strain engineering [1,2]. Compounds of elements from the 13th group of the periodic table, such as aluminium (Al), gallium (Ga), indium (In) and thallium (Tl), with nitrogen (N) are representatives of the MN family, and their 2D allotropes exhibit planar hexagonal graphene-like lattices [3,4]. For this reason, 2D aluminium nitride (AlN), gallium nitride (GaN), indium nitride (InN) and thallium nitride (TlN) nanostructures are envisioned to have superior physical and mechanical properties compared to those of their respective bulk counterparts [4,5]. Hexagonal aluminium nitride (h-AlN), gallium nitride (h-GaN) and indium nitride (h-InN) are wide-gap semiconductors and are capable of emitting light in green, blue and UV diapasons [6]. This makes h-AlN, h-GaN and h-InN promising materials in applications such as solid-state light-emitting diodes (LEDs) and field-effect transistors (FETs) [4,7,8]. On the other hand, hexagonal thallium nitride (h-TlN) has a small or rather negative energy band gap [5,9]. The latter denotes a material with a very small overlap between the bottom of its conduction band and the top of its valence band. Such materials are commonly known as semimetals, which is the case for h-TlN. This points to h-TlN being a suitable candidate for infrared optical devices [10]. Among 2D MNs, nanosheets (NSs) of aluminium nitride, gallium nitride and indium nitride have already been synthesized. The greatest success was achieved in the fabrication of AlN and GaN nanosheets. Aluminium nitride nanosheet (AlNNS) growth methods include chemical vapor deposition (CVD) [11], molecular beam epitaxy (MBE) [12], physical vapour transport (PVT) [13] and metal–organic chemical vapour deposition (MOCVD) [14]. Developments in the growth of gallium aluminium nanosheets (GaNNSs), in addition to CVD [15] and MOCVD [16] techniques, are owed to electrochemical etching (ECE) [17], UV-assisted electroless chemical etching [18] and ammonolysis of liquid metal-derived oxides [19]. The latter was also used to produce indium nitride nanosheets (InNNSs) [19]. The MBE [20] and MOCVD [21] techniques were also employed to synthesize InNNSs. Thallium nitride nanosheets (TlNNSs) have not yet been synthesized, and 2D TlN has only been the focus of computational investigations so far [4,5,10].

Moreover, in the work of Singh et al. [22], computational synthesis of hexagonal AlN, GaN and InN monolayers was suggested based on ab initio density functional theory (DFT) calculations. These results are helpful for establishing suitable synthesis conditions for 2D MNs, including identifying the best substrates for their growth and stabilization.

The key to accurate design and improved performance for innovative nanodevices and systems based on two-dimensional MNs is not only understanding the mechanical behaviour of their constituents but also its impact on the physical and chemical properties of these 2D MNs. Knowing the mechanical response of 2D MNs can ensure the durability and proper functioning of nanodevices containing nanosheets as building blocks. Additionally, strain engineering is an effective way to modify nanomaterials’ functional properties. From this perspective, the importance of evaluating the mechanical properties of metal nitride NSs increases significantly. As far as we know, the mechanical response of MN nanosheets has only been investigated theoretically up to now. To this end, most of the research has resorted to ab initio DFT calculations and molecular dynamics (MD), which are encompassed in atomistic approaches. The ab initio DFT method, which is suitable for a small number of atoms and requires substantial computational resources, was used in the works of Jafari et al. [23], Peng et al. [24], Kourra et al. [25] and Lv et al. [26] to assess the elastic properties of AlN nanosheets. Tuoc et al. [27] and Fabris et al. [28] employed the same approach to study the mechanical behaviour of GaN nanosheets, and Ahangari et al. [29] used it for the same type of study of AlNNSs and GaNNSs. Also, ab initio DFT calculations were employed in two works by Peng at al. [30,31] to assess the elastic constants of InNNSs [30] and TlNNSs [31]. Regarding the determination of the elastic properties of AlN, GaN and InN nanosheets, Luo et al. [32] and Faraji et al. [33] also used the ab initio DFT method. Although the ab initio DFT approach is established as a more accurate method than MD, the latter is more cost-effective for large atomic configurations. The MD method requires potential functions for modelling the interactions between the 13th group and nitrogen atoms, the choice of which, to a large extent, influences the results. Rouhi et al. [34] studied the mechanical response of GaN nanosheets, carrying out MD simulations employing the Tersoff–Brenner (TB) potential function. Singh et al. [35] determined the elastic constants of AlNNSs, GaNNSs and InNNSs, employing the TB potential to describe the interatomic interactions in the respective diatomic nanostructures. In an MD simulation study with Tersoff-like potentials, Le [36] evaluated the tensile properties of aluminium nitride, gallium nitride and indium nitride NSs. Sarma et al. [37] investigated the mechanical behaviour of GaN nanosheets using the Stillinger–Weber (SW) potential in their MD simulation study.

In addition to resource-consuming atomistic approaches, the nanoscale continuum modelling (NCM) method has been used to model the mechanical behaviour of 2D MNs. NCM, also known as the molecular structural mechanics (MSM) approach, has proven to be a fast and reliable method for evaluating the mechanical properties of 1D and 2D nanostructures with graphene and graphene-like lattices due to its simplicity and straightforward mathematical formulation in contrast to atomistic approaches (see, for example, [38,39,40,41]). The NCM/MSM approach is based on the linkage between the molecular structure and solid mechanics in such a way that the bonds connecting 13th group (Al, Ga, In or Tl) and N atoms are represented as elastic elements, most often beams or springs. Le [42] derived a closed-form solution within the NCM/MSM method for calculating the Young’s modulus of aluminium nitride NSs. Ben et al. [2] assessed the maximum stress and tensile strain of AlNNSs, GaNNSs and InNNSs using the respective closed-form expressions within the scope of the NCM/MSM method. Using the same approach, Giannopoulos et al. [43] modelled the interatomic bonding between Ga and N atoms as spring elements to study the tensile behaviour of GaN nanosheets. In two of their studies, Sakharova et al. [44,45], in the framework of the NCM/MSM method, represented interatomic bonds as beam elements to evaluate the Young’s modulus of square InNNSs [44] and the Young’s and shear moduli of AlNNSs and GaNNSs over a wide range of aspect ratios [45].

It is worth noting that there is a certain inconsistency in the elastic properties of 2D MNs reported in the literature. Also, it can be reckoned that research on the mechanical behaviour of 2D metal nitrides has not been systematized to date and has focused mainly on AlNNSs and GaNNSs, while studies that include the other two representatives of MNs have been less frequent (InNNSs) or very rare (TlNNSs).

The aim of the current work was to carry out a systematic comparative study on the evaluation of the surface Young’s and shear moduli and Poisson’s ratio of aluminium nitride, gallium nitride, indium nitride and thallium nitride nanosheets (AlNNSs, GaNNSs, InNNSs and TlNNSs). With this purpose, a three-dimensional finite element (FE) model was developed under the NCM/MSM approach. The latter was chosen as a fast and cost-effective method for modelling the elastic behaviour of nanostructures. The NCM/MSM method does not require significant computational resources and can be effectively used for massive atomic structures, unlike atomistic methods. In view of the lack of information on the thickness values of MN nanosheets, the surface elastic moduli were selected for analysis in this study. It is expected that the present study will contribute to our knowledge of the mechanical response of two-dimensional metal nitrides and unlock new perspectives on their applications in novel nanodevices.

## 2. Materials and Methods

### 2.1. Modelling of the Elastic Behaviour of MN Nanosheets

The bonds between atoms of the diatomic MN lattices were modelled as equivalent beam elements within the scope of the NCM/MSM approach. The resulting equivalent continuum structure is characterized by the tensile, $E_{b}A_{b}$; bending, $E_{b}I_{b}$; and torsional, $G_{b}J_{b}$, rigidities of the beams, which are linked to the molecular structure of the nanosheet, through bond stretching, $k_{r}$; bond bending, $k_{\theta }$; and torsional resistance, $k_{\tau }$, force field constants as follows [46]:

(1) $$E_{b}A_{b}=lk_{r},\;E_{b}I_{b}=lk_{\theta },\;G_{b}J_{b}=lk_{\tau }$$

where $A_{b}=\pi d^{2}/4$ is the cross-section area, $I_{b}=\pi d^{4}/64$ is the moment of inertia, $J_{b}=\pi d^{4}/32$ is the polar moment of inertia of the beam element, which has a circular cross-section and diameter d, and *l* is the beam length, equal to the bond length of the diatomic metal nitride nanostructures, $a_{M\text{-}N}$.

Equation (1) permit calculation of the input parameters for the numerical simulation utilizing the $k_{r}$, $k_{\theta }$ and $k_{\tau }$ force field constants. For the metal nitrides under study, the bond stretching, $k_{r}$, and bond bending, $k_{\theta }$, force constant values are scarce in the literature. Consequently, in the current work, the bond stretching and bond bending force constants are assessed using a method based on analytical expressions from the molecular mechanics (MM) for the surface Young’s modulus, $E_{s}$, and Poisson’s ratio, ν. The $E_{s}$ and ν, values can be obtained using DFT calculations or experimentally. Both the $k_{r}$ and $k_{\theta }$ force constants are related to $E_{s}$ and ν through the following expressions [47]:

(2) $$\left\{\begin{array}{c} E_{s}=\frac{4\sqrt{3}k_{r}k_{\theta }}{k_{r}\frac{a_{M\text{-}N}^{2}}{2}+9k_{\theta }}\\ \nu =\frac{k_{r}a_{M\text{-}N}^{2}-6k_{\theta }}{k_{r}a_{M\text{-}N}^{2}+18k_{\theta }}. \end{array}\right.$$

The bond stretching, $k_{r}$, and bond bending, $k_{\theta }$, force constants are assessed by solving the system of Equation (2) as follows:

(3) $$k_{r}=\frac{3E_{s}}{\sqrt{3}\left(1-\nu \right)}$$

(4) $$k_{\theta }=\frac{E_{s}a_{M\text{-}N}^{2}}{2\sqrt{3}\left(1+3\nu \right)}$$

The bond length, $a_{M\text{-}N}$; the surface Young’s modulus, $E_{s}$; and Poisson’s ratio, ν required to compute the bond stretching, $k_{r}$, and bending, $k_{\theta }$, force field constants (Equations (3) and (4)), together with their calculated values, are shown in Table 1. The values of $E_{s}$ and ν used in Equations (3) and (4) were taken from the DFT calculation results obtained by Şahin et al. [3] for AlN, GaN and InN and by Ye and Peng [4] for TlN. The torsional resistance force field constant, $k_{\tau }$, was obtained basing on the DREIDING force field [48], which allowed us to describe the torsional behaviour of the diatomic nanostructure only on the basis of the hybridization of the atoms. The value of $k_{\tau }$ is also displayed in Table 1.

Knowledge of the $k_{r}$, $k_{\theta }$, and $k_{\tau }$ values (Table 1) permitted us to calculate the geometrical and elastic properties (the input values for the numerical simulation) of the beams using Equation (1) assuming that $a_{M\text{-}N}$ = *l*, as shown in Table 2.

### 2.2. Finite Element Analysis and Elastic Properties of MN Nanosheets

Square single-layer AlNNSs, GaNNSs, InNNSs and TlNNSs with dimensions ≈15 × 15 nm^2^ were studied. This size of the NSs was chosen to ensure that their mechanical response was independent of their size since the elastic properties of square nanosheets have been shown to be nearly constant with increasing their side lengths, with the exception of a range of small NSs [45,49]. The finite element (FE) meshes of the MN nanosheets were obtained in the form of Program Database files using the Nanotube Modeler© software (version 1.8.0, ©JCrystalSoft, http://www.jcrystal.com, accessed on 1 September 2024). The bond lengths for the FE meshes of the AlNNSs and GaNNSs, $a_{Al\text{-}N}\;$= 0.183 nm and $a_{Ga\text{-}N}\;$= 0.195 nm, respectively, were assumed as defined by the Nanotube Modeler© program. For the InNNSs and TlNNSs, bond lengths $a_{In\text{-}N}\;$= 0.206 nm [3] and $a_{Tl\text{-}N}\;$= 0.2154 nm [4] were adopted, respectively. The next step was to convert the Program Database files into a format usable by ABAQUS^®^ (Abaqus 2020, Dassault Systèmes^®^) FE code, resorting to the in-house application InterfaceNanosheets.NS [49]. Afterwards, the abovementioned code was utilized to perform finite element analysis (FEA) of the elastic response of the MN nanosheets under numerical tensile and in-plane shear tests.

**Figure 1:**
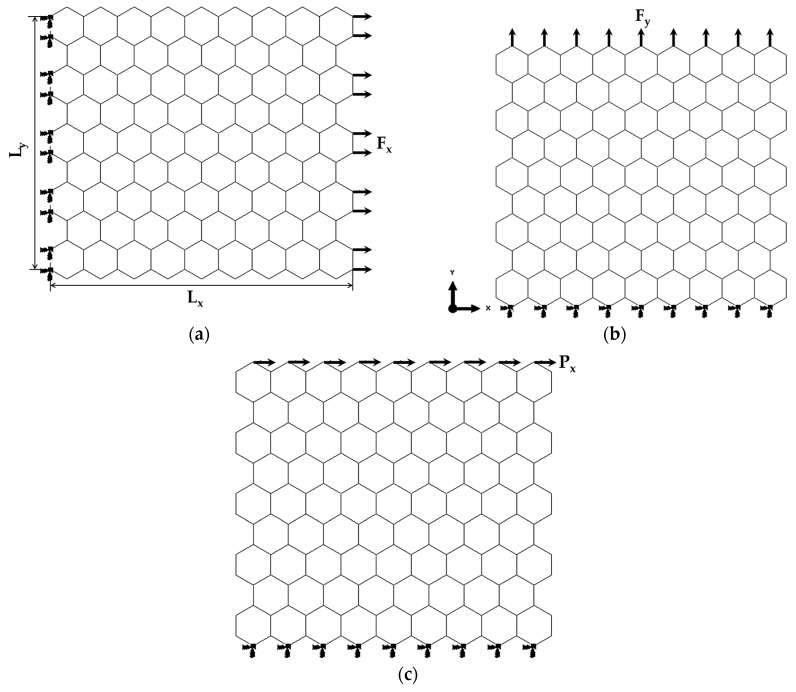
Schematic representation of the loading and boundary conditions for the TlNNS: (**a**) tensile loading in the x-direction (zigzag configuration); (**b**) tensile loading in the y-direction (armchair configuration); and (**c**) in-plane shear loading in the x-direction.

To simulate the elastic behaviour of the NSs in the x-direction, an axial tensile load, $F_{x}$, was applied to the nodes on the right side of the NSs, leaving the opposite side fixed (Figure 1a). The Young’s modulus along the x-axis, $E_{x}$, is determined as follows [40]:

(5) $$E_{x}=\frac{F_{x}L_{x}}{u_{x}L_{y}t_{n}}$$

where $u_{x}$ is the NS’s axial displacement (elongation in the x-direction) taken from the FEA; $L_{x}$ and $L_{y}$ are the NS’s side lengths (see Figure 1a); and $t_{n}$ is the nanosheet’s thickness.

In turn, based on the results of the tensile test used to assess $E_{x}$, Poisson’s ratio, $\nu_{\mathrm{xy}}$, is evaluated as follows [40]:

(6) $$\nu_{\mathrm{xy}}=\frac{u_{y}L_{x}}{{u_{x}L}_{y}}$$

where $u_{y}$ is the transversal displacement measured in the FEA, at $x=L_{x}/2$.

Similarly, to simulate tension in the y-direction, an axial force, $F_{y}$, was applied to the edge nodes on the upper side of the NS, leaving the lower side fixed (Figure 1b). The Young’s modulus along the y-axis, $E_{y}$, is determined as follows [40]:

(7) $$E_{y}=\frac{F_{y}L_{y}}{v_{y}L_{x}t_{n}}$$

where $v_{y}$ is the NS axial displacement in the y-direction, taken from the FEA.

The two tensile loading conditions for the MN nanosheets, as shown in Figure 1a and b, represent zigzag and armchair configurations, respectively.

To simulate the in-plane shear test, a shear load $P_{x}$ was applied to the upper side of the NS, leaving the edge nodes of the bottom side of the NS fixed (Figure 1c). Consequently, the NS’s shear modulus, $G_{\mathrm{xy}}$, is calculated as follows [40]:

(8) $$G_{\mathrm{xy}}=\frac{P_{x}}{\gamma_{\mathrm{xy}}L_{x}t_{n}},\;\gamma_{\mathrm{xy}}=tan\frac{s_{x}}{L_{y}}$$

where $s_{x}$ is the displacement along the x-axis, taken from the FEA and measured in the central part of the nanosheet; $L_{x}$ and $L_{y}$ are the side lengths of the NS (see Figure 1a); and $t_{n}$ is the NS’s thickness.

In the current study, assuming a lack of information in relation to the value of $t_{n}$ for the MNs, the surface Young’s and shear moduli $E_{sx}$ and $E_{\mathrm{sy}}$ and $G_{\mathrm{sxy}}$ (the product of the respective elastic moduli and the NS’s thickness) were calculated instead of $E_{x}$, $E_{y}$ and $G_{\mathrm{xy}}$. To this end, Equations (6)–(8) were transformed as follows:

(9) $${E_{sx}=E}_{x}t_{n}=\frac{F_{x}L_{x}}{{u_{x}L}_{y}}$$

(10) $$E_{\mathrm{sy}}=E_{y}t_{n}=\frac{F_{y}L_{y}}{v_{y}L_{x}}$$

(11) $$G_{\mathrm{sxy}}=G_{\mathrm{xy}}t_{n}=\frac{P_{x}}{\gamma_{\mathrm{xy}}L_{x}}$$

## 3. Results and Discussion

### 3.1. Surface Young’s Moduli and Poisson’s Ratio of the MN Nanosheets

**Figure 2:**
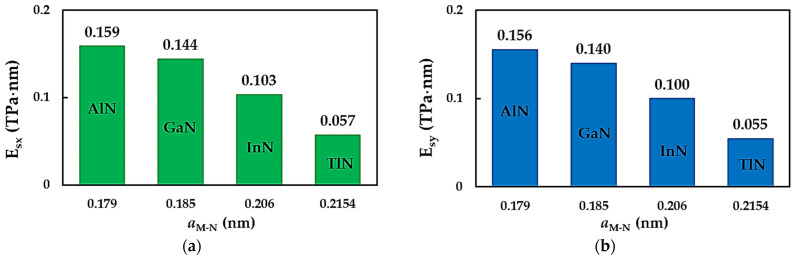
The surface Young’s moduli, as a function of the bond length $a_{M\text{-}N}$, of the diatomic NS structure for (**a**) the zigzag configuration, $E_{\mathrm{sx}}$, and (**b**) the armchair configuration, $E_{\mathrm{sy}}$, of the MN nanosheets.

**Figure 3:**
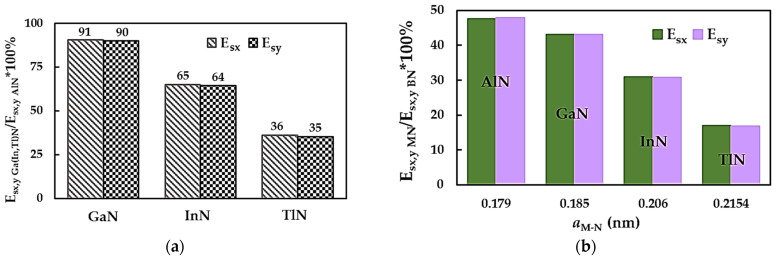
Comparison of the surface Young’s moduli $E_{\mathrm{sx},y}$ of (**a**) GaNNSs, InNNSs and TlNNSs with those of AlNNSs and (**b**) those of metal nitride NSs with those of BNNSs [49].

The surface Young’s moduli of the metal nitride NSs in the x-direction (zigzag configuration), $E_{\mathrm{sx}}$, and in the y-direction (armchair configuration), $E_{\mathrm{sy}}$, were calculated using Equations (9) and (10), respectively, making use of the tensile simulation results. The values of $E_{\mathrm{sx}}$ and $E_{\mathrm{sy}}$ as a function of the bond length, $a_{M\text{-}N}$, are shown in Figure 2a and b, respectively, for the AlN, GaN, InN and TlN nanosheets. The surface Young’s moduli, $E_{\mathrm{sx},y}$, of the 2D MNs decreases with an increasing value of $a_{M\text{-}N}$. The smaller the interatomic bond length, the higher the value of $E_{\mathrm{sx},y}$. Thus, the highest surface Young’s modulus is observed for NSs made of AlN. The average $E_{\mathrm{sx},y}$ values for the GaNNSs, InNNSs and TlNNSs are approximately 90%, 65% and 36%, respectively, of those calculated for the AlNNSs (see Figure 3a). In order to understand how to use metal nitride monolayers in the construction of novel nanodevices best, the surface Young’s moduli, $E_{\mathrm{sx},y}$, of the AlNNSs, GaNNSs, InNNSs and TlNNSs, normalized by those for boron nitride nanosheets (BNNSs), are plotted in Figure 3b. Boron (B) is a non-metal that belongs to the 13th group of the periodic table, like the metals Al, Ga, In and Tl, and hexagonal boron nitride (h-BN) is an insulator with remarkable mechanical properties, similar to those of graphene [50,51]. The surface Young’s moduli of the BNNSs in the zigzag and armchair arrangements, calculated from the Young’s modulus results from Sakharova et al. [49], $E_{\mathrm{sx}}$ = 0.334 TPa·nm and $E_{\mathrm{sy}}$ = 0.324 TPa·nm, respectively, were selected for comparison purposes. The BNNSs’ Young’s modulus values from the work [49] were assessed using the same calculation method as in the current study and were comparable or in reasonable agreement (at a difference of 5.4%) with those obtained experimentally [49]. This may indicate the reliability of the present Young’s modulus results for MN nanosheets.

As shown in Figure 3b, the $E_{\mathrm{sx},y}$ values of the AlNNSs, GaNNSs, InNNSs and TlNNSs are about 48%, 43%, 31% and 17%, respectively, of the BNNSs’ surface Young’s moduli. Even the most mechanically resistant in the MN group, the aluminium nitride NSs, have $E_{\mathrm{sx},y}$ values that are almost two times lower than those of boron nitride NSs. This must be taken into consideration when developing novel applications involving MN monolayers. To take advantage of the electronic, optical and thermal properties of 2D metal nitrides better without compromising the robustness and operation of nanodevices and systems, MN nanosheets, especially those with weaker tensile properties such as InNNSs and TlNNSs, should be combined with, for example, BNNSs or graphene. It is worth noting that the surface Young’s moduli of 2D nanostructures formed of 13th group–nitride compounds are close to those of their 1D counterparts, i.e., nanotubes (NTs) [52]. Thus, both 1D and 2D allotropes can be exploited in the design and manufacturing of innovative nanodevices without them losing their strength and durability.

**Figure 4:**
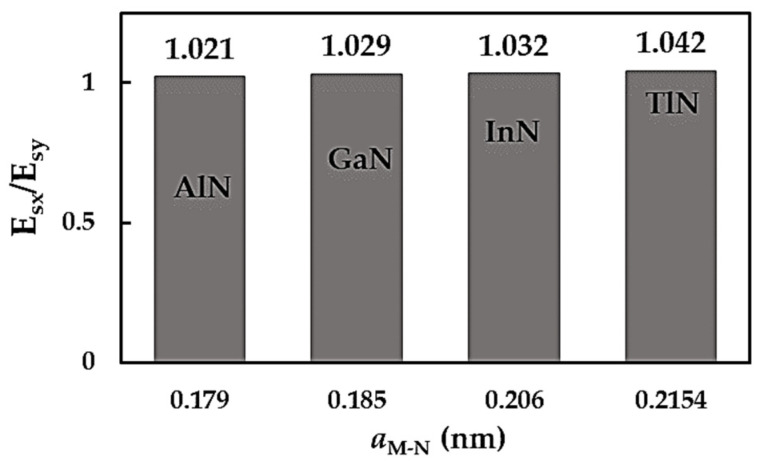
Ratio between the surface Young’s moduli in the zigzag and armchair directions, $E_{\mathrm{sx}}/E_{\mathrm{sy}}$, as a function of the bond length, $a_{M\text{-}N}$, for MN nanosheets.

It can be observed that the surface Young’s modulus of the MN nanosheets is to some extent higher for the zigzag configuration than for the armchair configuration, $E_{\mathrm{sx}}$ > $E_{\mathrm{sy}}$, which indicates the anisotropy of the AlNNSs, GaNNSs, InNNSs and TlNNSs. In a previous study by the authors [49], such anisotropic behaviour was reported for BNNSs and was explicated by the different stresses required for the elongation of the hexagonal lattice in the x- and y-directions; this is because the atomic arrangement for a zigzag configuration differs from that in an armchair configuration with respect to the axial load applied. The anisotropic behaviour of an NS can be quantified by the ratio between its surface Young’s moduli in the zigzag and armchair directions, $E_{\mathrm{sx}}/E_{\mathrm{sy}}$. The evolution of the $E_{\mathrm{sx}}/E_{\mathrm{sy}}$ ratio for the 2D MN nanostructures with their bond length, $a_{M\text{-}N}$, is shown in Figure 4.

The $E_{\mathrm{sx}}/E_{\mathrm{sy}}$ ratio increases from 1.021 (AlNNSs) to 1.042 (TlNNSs) with an increasing bond length, $a_{Al\text{-}N}$ = 0.179 nm < $a_{Ga\text{-}N}$ = 0.185 nm < $a_{In\text{-}N}$ = 0.206 nm < $a_{Tl\text{-}N}$ = 0.215 nm. It can be concluded that the metal nitride nanosheets exhibit mild anisotropy regardless of the compound that forms the 2D MN nanostructure.

**Figure 5:**
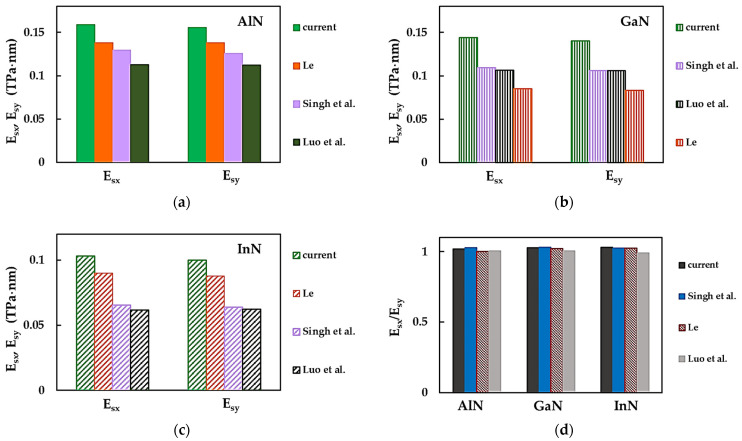
Comparison of the current surface Young’s moduli, $E_{\mathrm{sx},y}$, of (**a**) AlNNSs, (**b**) GaNNSs and (**c**) InNNSs and (**d**) $E_{\mathrm{sx}}/E_{\mathrm{sy}}$ ratios for the MNs from (**a**–**c**) with those available in the literature [32,35,36].

For comparison purposes, the present surface Young’s moduli values, $E_{\mathrm{sx}}$ and $E_{\mathrm{sy}}$, and their ratios, $E_{\mathrm{sx}}/E_{\mathrm{sy}}$, together with the respective results from the literature, are plotted in Figure 5 for AlN, GaN and InN nanosheets. Reasonable concordance (at a difference ≈ 14%) is observed when the $E_{\mathrm{sx},y}$ values calculated in the present study for AlNNSs and InNNSs are compared with those reported by Le [36], who used the analytical expression obtained within the NCM/MSM method. The surface Young’s moduli as assessed by Singh et al. [35] for GaNNSs and InNNSs are in a very good agreement with these respective $E_{\mathrm{sx},y}$ values as assessed by Luo et al. [32] (see Figure 5b,c). To this end, Singh et al. [35] employed MD simulations with the TB potential function to describe the interactions between Ga (In) and N atoms, while Luo et al. [32] used ab initio DFT calculations.

Regarding the ratio between the surface Young’s moduli in the zigzag and armchair directions, Le [36] for AlNNSs and Luo et al. [32] for AlNNSs and GaNNSs found that $E_{\mathrm{sx}}/E_{\mathrm{sy}}$ ≈ 1, which suggests the isotropic behaviour of these MN nanosheets (see Figure 5d). On the other hand, Le [36] reported anisotropic behaviour for GaNNSs and InNNSs and Luo et al. [32] for InNNSs. In the latter case, a ratio $E_{\mathrm{sx}}/E_{\mathrm{sy}}$ < 1 applies. According to Singh et al. [35], the AlNNSs, GaNNSs and InNNSs under study are transversely anisotropic. For all metal nitride NSs in Figure 5d, which demonstrate anisotropic behaviour, except for the InNNSs studied by Luo et al. [35], the surface Young’s modulus in the zigzag direction is slightly higher than that in the armchair direction, $E_{\mathrm{sx}}$ > $E_{\mathrm{sy}}$, i.e., $E_{\mathrm{sx}}/E_{\mathrm{sy}}$ > 1. The current $E_{\mathrm{sx}}/E_{\mathrm{sy}}$ ratios for aluminium nitride, gallium nitride and indium nitride NSs are in a good agreement (the biggest difference of 0.87%) with those reported in the literature, meaning there is mild anisotropy of the nanosheets in the transversal direction.

**Figure 6:**
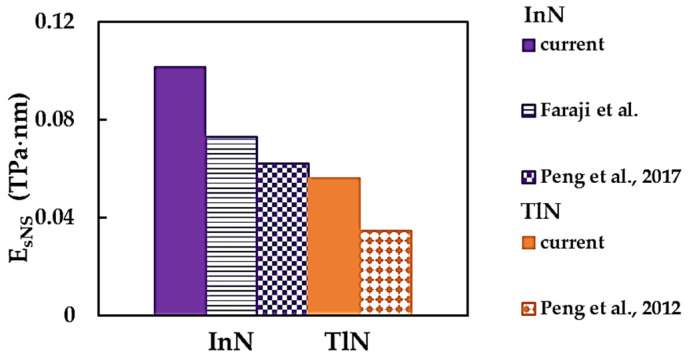
Comparison of the present surface Young’s moduli, $E_{\mathrm{sNS}}$, for InNNSs and TlNNSs with those from the studies of Faraji et al. [33] and Peng et al. (2012, 2017) [30,31].

Figure 6 compares the present average values for the surface Young’s modulus, calculated by $E_{\mathrm{sNS}}=\left(E_{\mathrm{sx}}+E_{\mathrm{sy}}\right)/2$, for the InNNSs and TlNNSs with those reported by Peng et al. [30,31]. The choice of InN and TlN nanosheets was due to the fact that a comprehensive comparison of $E_{\mathrm{sNS}}$ moduli for AlNNSs and GaNNSs with the results available in the literature was performed by the authors in previous work [45].

The currently used NCM/MSM approach leads to higher $E_{\mathrm{sNS}}$ values for InNNSs and TlNNSs when compared with the respective results from the works [30,31,33]. Faraji et al. [33] and Peng et al. [30] assessed the surface Young’s modulus of InNNSs, resorting to the Vienna ab initio simulation package (VASP) for their ab initio DFT calculations. Both studies implemented the generalized gradient approximation (GGA) as parameterized by the Perdew–Burke–Ernzerhof (PBE) functional to describe the exchange–correlation energy. Although the calculation approach is similar, it leads to different surface Young’s modulus results for InNNSs. Figure 5a–c and Figure 6 show a noticeable scattering of the surface Young’s modulus values for the MN nanosheets, as well as a lack of results, especially for thallium nitride NSs.

**Figure 7:**
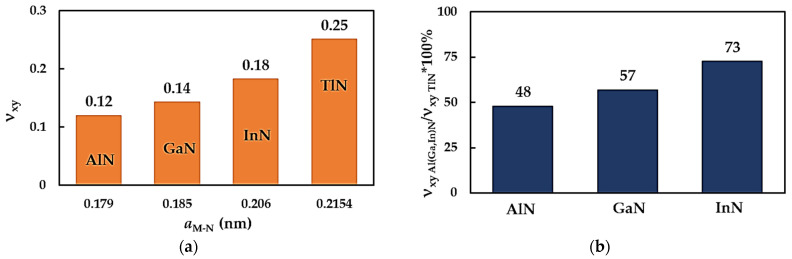
(**a**) Evolution of the Poisson’s ratio, $\nu_{\mathrm{xy}}$, of MN nanosheets as a function of the bond length, $a_{M\text{-}N}$; (**b**) comparison of the $\nu_{\mathrm{xy}}$ values for AlNNSs, GaNNSs and InNNSs with those of TlNNSs.

The metal nitrides NSs’ Poisson’s ratios, $\nu_{\mathrm{xy}}$, calculated with the help of Equation (6), are shown in Figure 7a as a function of the diatomic structure bond length, $a_{M\text{-}N}$. The value of $\nu_{\mathrm{xy}}$ for the metal nitride NSs increases nearly two times over, from 0.12 (AlNNSs) to 0.25 (TlNNSs), with an increase in $a_{M\text{-}N}$. The Poisson’s ratio for the AlNNSs, GaNNSs and InNNSs consists of about 48%, 57% and 73%, respectively, of the value of $\nu_{\mathrm{xy}}$ obtained for the TlNNSs, as displayed in Figure 7b.

**Figure 8:**
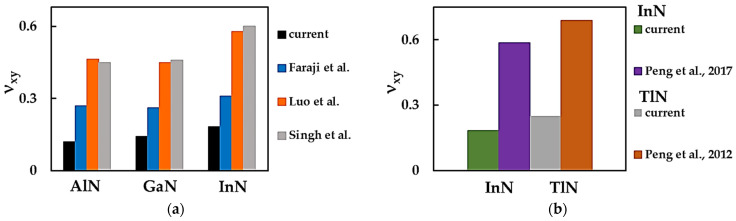
Comparison of the Poisson’s ratio, $\nu_{\mathrm{xy}}$, of (**a**) AlNNSs, GaNNSs and InNNSs abd (**b**) InNNSs and TlNNSs with the respective values from the literature [30,31,32,33,35].

Figure 8 compares the current Poisson’s ratio results with those from the literature for the MN nanosheets. The values of $\nu_{\mathrm{xy}}$ calculated in the present study for AlNNSs, GaNNSs, InNNSs and TlNNSs are considerably lower than those evaluated by Luo et al. [32], Singh et al. [35], Faraji et al. [33] and Peng et al. [30,31].

Good agreement is observed between the $\nu_{\mathrm{xy}}$ values obtained by Luo et al. [32] and Singh et al. [35], with differences of ≈2.9%, 2.2% and 3.7% for AlNNSs, GaNNSs and InNNSs, respectively. In both studies, an atomistic approach was used, although Luo et al. [32] calculated Poisson’s ratio by employing the VASP within the ab initio DFT method and GGA-PBE for the exchange–correlation energy, and Singh et al. [35] used MD simulations with the TB potential to this end. It is worth noting that Faraji et al. [33], who used the same calculation methodology as Luo et al. [32], obtained values of $\nu_{\mathrm{xy}}$ ≈ 58%, 58% and 54% of those obtained by Luo et al. [32] for the corresponding AlN, GaN and InN nanosheets.

Despite the Poisson’s ratio values obtained by Luo et al. [32], Singh et al. [35] and Faraji et al. [33] for AlN, GaN and InN nanosheets being different from those currently computed, the trends in the evolution of $\nu_{\mathrm{xy}}$ with the bond length $a_{M\text{-}N}$ are comparable. As seen in Figure 8a, the values of $\nu_{\mathrm{xy}}$ for the AlNNSs, GaNNSs and InNNSs obtained by Luo et al. [32], Singh et al. [35] and Faraji et al. [33] increase when $a_{M\text{-}N}$ increases, although the Poisson’s ratios for the AlNNSs and GaNNSs are similar. This can be explained by the close values for the bond length, $a_{Al\text{-}N}$ and $a_{Ga\text{-}N}$, used in these studies [32,33,35].

It can be concluded from Figure 8 that for metal nitride NSs, there is a scarcity and wide spread of Poisson’s ratio values. Considerably more $\nu_{\mathrm{xy}}$ results are necessary to establish a reliable benchmark for ascertaining this elastic property using analytical and numerical methods.

### 3.2. Surface Shear Moduli of the MN Nanosheets

**Figure 9:**
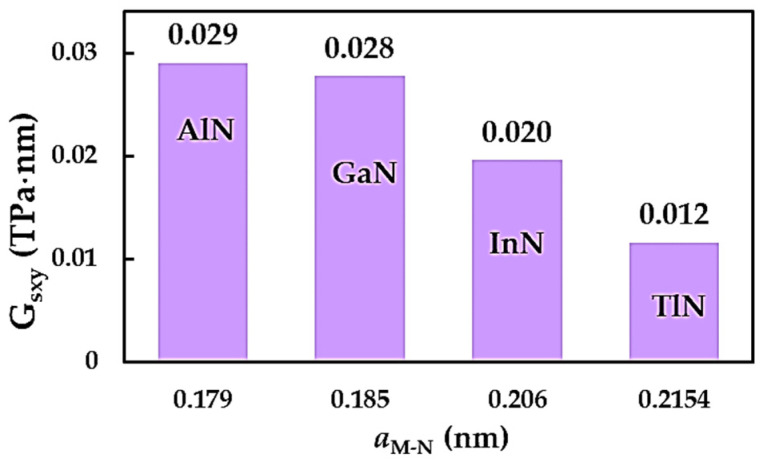
Evolution of the surface shear moduli, $G_{\mathrm{sxy}}$, of the MN nanosheets as a function of the respective bond length, $a_{M\text{-}N}$.

The evolution of the surface shear moduli, $G_{\mathrm{sxy}}$, for AlNNSs, GaNNSs, InNNSs and TlNNSs, computed with the aid of Equation (11), as a function of the respective bond length, $a_{M\text{-}N}$, is shown in Figure 9. $G_{\mathrm{sxy}}$ decreases to a degree ranging from 0.029 TPa·nm (AlNNSs) to 0.012 TPa·nm (TlNNSs) when the value of $a_{M\text{-}N}$ increases.

**Figure 10:**
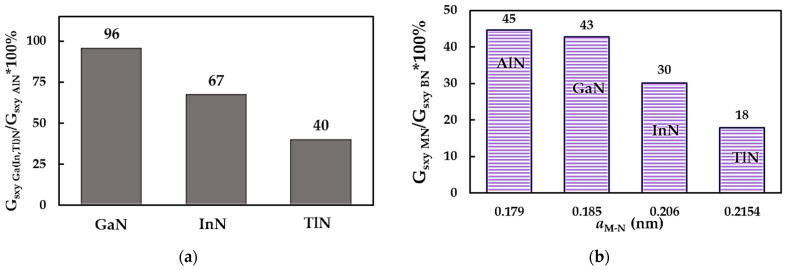
Comparison of the surface shear moduli, $G_{\mathrm{sxy}}$, of (**a**) GaNNSs, InNNSs and TlNNSs with those of AlNNSs and (**b**) of the metal nitride NSs with those of BNNSs [49].

Figure 10a facilitates a comparison of the surface shear modulus results, $G_{\mathrm{sxy}}$, for the metal nitride NSs under study by normalizing those for the GaNNSs, InNNSs and TlNNSs by that for the AlNNSs, which have the greatest $G_{\mathrm{sxy}}$ values among the MN group. The surface shear modulus of GaN, InN and TlN nanosheets is about 96%, 67% and 40%, respectively, of $G_{\mathrm{sxy}}$ for the aluminium nitride NSs. As can be noticed from the results displayed in Figure 10a, the surface shear moduli of AlNNSs and GaNNSs are close in value.

**Figure 11:**
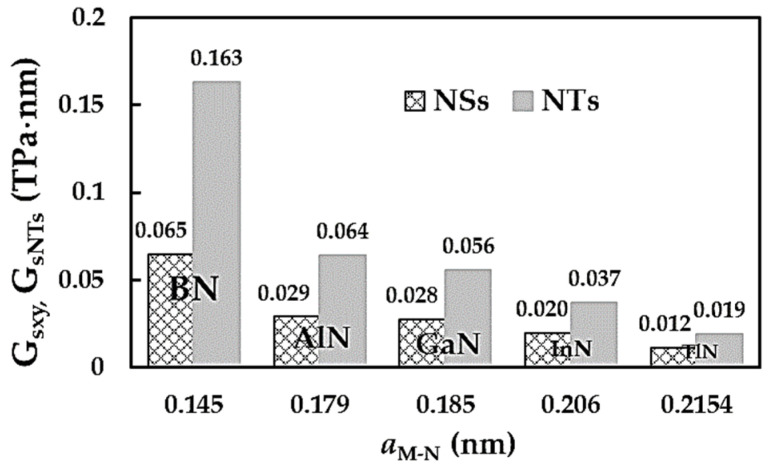
Comparison of the surface shear moduli, $G_{\mathrm{sxy}}$, of the 13th group–nitride NSs with the shear moduli, $G_{\mathrm{sNTs}}$, of NTs of the same materials [52]. $G_{\mathrm{sxy}}$ and $G_{\mathrm{sNTs}}$ values for BN nanosheets and nanotubes are from [39,49], respectively.

Similar to the surface Young’s modulus results for the MN nanosheets (see Figure 3b), their surface shear moduli were compared with those of boron nitride NSs, as shown in Figure 10b. $G_{\mathrm{sxy}}$ as calculated for AlNNSs, GaNNSs, InNNSs and TlNNSs is ≈45%, 43%, 30% and 18%, respectively, of the values for BNNSs’ surface shear moduli. To understand the present results on the surface shear modulus $G_{\mathrm{sxy}}$ better, the values for the metal nitride NSs are plotted together with the surface shear modulus for the respective NTs, $G_{\mathrm{sNTs}}$, in Figure 11. The values for the $G_{\mathrm{sNTs}}$ were taken from previous work by the authors [52] and, similar to the current study, were obtained by resorting to numerical simulation under the NCM/MSM method. To complete the comparison, the $G_{\mathrm{sxy}}$ and $G_{\mathrm{sNTs}}$ values for boron nitride NSs [49] and NTs [39] are also plotted in Figure 11.

In contrasting the surface Young’s moduli of the NTs and NSs of the 13th group–nitride compounds (see Section 3.1), the nanotubes’ surface shear moduli are 2.5, 2.2, 2.0, 1.9 and 1.6 times bigger than the values of $G_{\mathrm{sxy}}$ for boron nitride, aluminium nitride, gallium nitride, indium nitride and thallium nitride nanosheets, respectively. It can be concluded that NSs (2D nanostructures) based on these nitride compounds have inferior shear properties when compared to their 1D (NT) counterparts. This should be taken into account in the design of nanodevices and systems, where higher mechanical resistance of the constituents to the applied shear stress is required.

**Figure 12:**
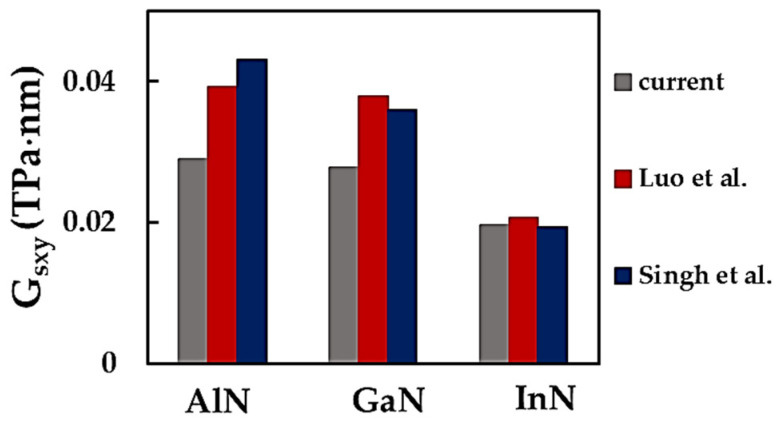
Comparison of the current surface shear modulu, $G_{\mathrm{sxy}}$, of AlNNSs, GaNNSs and InNNSs [32,35].

To the best of our knowledge, results on the surface shear modulus of MN nanosheets are scarce or even non-existent (in the case of TlNNSs) in the literature. Figure 12 compares the current values of the surface shear modulus for AlNNSs, GaNNSs and InNNSs with those from the works by Luo et al. [32] and Singh et al. [35].

The $G_{\mathrm{sxy}}$ value calculated in the present study for InNNSs shows very good concordance when compared to those reported by Singh et al. [35] and Luo et al. [32], with respective differences of about 0.9% and 5.6%. For AlNNSs and GaNNSs, the current value of $G_{\mathrm{sxy}}$ is considerably lower (in the range of 30% to 49%) than those obtained by Singh et al. [35] and Luo et al. [32]. In these studies, the differences between the surface shear moduli were 10.0%, 4.8% and 6.1% for AlN, GaN and InN nanosheets, respectively. A decreasing trend in the evolution of the surface shear modulus with an increasing bond length is observed in the current work, similar to the results reported by Singh et al. [35] and Luo et al. [32] (see Figure 12). In short, the scarcity of $G_{\mathrm{sxy}}$ values in the literature to date does not allow pertinent conclusions to be drawn with regard to the mechanical response of the metal nitride NSs under shear loading. Furthermore, more shear modulus results are required to establish a reference for ascertaining the shear elastic properties of MN nanosheets using theoretical approaches. The present study has attempted to fill this gap.

## 4. Conclusions

In the current work, the elastic properties, viz. the surface Young’s and shear moduli and the Poisson’s ratio, of 2D metal nitrides with graphene-like lattices (AlNNSs, GaNNSs, InNNSs and TlNNSs) were evaluated based on the NCM/MSM method. To the best of our knowledge, this was the first time a systematic comparative study of this kind, which comprised all metals of the 13th group of the periodic table, was performed. The main conclusions are given below.

The surface Young’s and shear moduli and the Poisson’s ratios of AlN, GaN, InN and TlN nanosheets are sensitive to the bond length of the honeycomb diatomic arrangement. The surface Young’s and shear moduli decrease, whereas the Poisson’s ratio increases, with an increase in the interatomic bond length.

The surface Young’s modulus of AlNNSs, GaNNSs, InNNSs and TlNNSs is at least half of that obtained for boron nitride or graphene nanosheets. This result should be taken into consideration during the design of prospective complex systems and nanodevices for which 2D metal nitride nanostructures are considered potential constituents.

It was shown that the surface shear modulus of the 2D nitride nanostructures (NSs) was about two times lower than that observed for their 1D counterparts (NTs), indicating the weaker mechanical properties of the nitride nanosheets under in-plane shear loading.

The results achieved represent substantial input into the knowledge and determination of the elastic properties of metal nitride nanosheets using analytical and numerical approaches.

## Figures and Tables

**Table 1:** Bond length, surface Young’s modulus, Poisson’s ratio and $k_{r}$, $k_{\theta }$ and $k_{\tau }$ force field constants for AlN, GaN, InN and TlN nanosheets.

Compound	$a_{M\text{-}N}$, nm [3]	E_s_, nN/nm [3]	ν [3]	$k_{r}$, nN/nm	$k_{\theta }$, nN·nm/rad^2^	$k_{\tau }$, nN·nm/rad^2^
AlN	0.179	116	0.46	372	0.451	0.625
GaN	0.185	110	0.48	366	0.445	
InN	0.206	67	0.59	283	0.296	
TlN	0.2154 *	34.5 *	0.689 *	192	0.151	

* Values from Ye and Peng [4].

**Table 2:** Geometrical and elastic properties of the beams, together with their respective formulations, as input parameters for numerical simulation.

Compound	Diameter, d, nm	Formulation	Young’s Modulus, E_b_, GPa	Formulation	Shear Modulus, G_b_, GPa	Formulation	Poisson’s Ratio, $\nu_{b\;}$
AlN	0.1392	$d=4\sqrt{\frac{k_{\theta }}{k_{r}}}$	4374	$E_{b}=\frac{k_{r}^{2}l}{4\pi k_{\theta }}$	3032	$G_{b}=\frac{{k_{r}^{2}k}_{\tau }l}{8\pi \;k_{\theta }^{2}}$	0.46 [3]
GaN	0.1395		4437		3113		0.48 [3]
InN	0.1294		4432		4674		0.59 [3]
TlN	0.1120		4200		8712		0.689 [4]

## Data Availability

The data presented in this study are available on request from the corresponding author after obtaining the permission of the authorized person.

## References

[1] Zheng F., Xiao X., Xie J., Zhou L., Li Y., Dong H. (2022). Structures, properties and applications of two-dimensional metal nitrides: From nitride MXene to other metal nitrides. 2D Mater..

[2] Ben J., Liu X., Wang C., Zhang Y., Shi Z., Jia Y., Zhang S., Zhang H., Yu W., Li D. (2021). 2D III-Nitride Materials: Properties, Growth, and Applications. Adv. Mater..

[3] Şahin H., Cahangirov S., Topsakal M., Bekaroglu E., Akturk E., Senger R.T., Ciraci S. (2009). Monolayer honeycomb structures of group-IV elements and III-V binary compounds: First-principles calculations. Phys. Rev. B.

[4] Ye C., Peng Q. (2023). Mechanical Stabilities and Properties of Graphene-like 2D III-Nitrides: A Review. Crystals.

[5] Elahi S.M., Farzan M., Salehi H., Abolhasani M.R. (2016). An investigation of electronic and optical properties of TlN nanosheet and compare with TlN bulk (Wurtzite) by first principle. Optik.

[6] Vurgaftman I., Meyer J.R. (2003). Band parameters for nitrogen-containing semiconductors. J. Appl. Phys..

[7] Wu K., Huang S., Wang W., Li G. (2021). Recent progress in III-nitride nanosheets: Properties, materials and applications. Semicond. Sci. Technol..

[8] Wang Z., Wang G., Liu X., Wang S., Wang T., Zhang S., Yu J., Zhao G., Zhang L. (2021). Two-dimensional wide band-gap nitride semiconductor GaN and AlN materials: Properties, fabrication and application. J. Mater. Chem. C.

[9] Zaoui A. (2003). Plane wave pseudopotential study of ground state properties and electrochemical description of thallium nitride. Mater. Sci. Eng. B.

[10] Abdullah N.R., Abdullah B.J., Gudmundsson V. (2022). Electronic and optical properties of metallic nitride: A comparative study between the MN (M = Al, Ga, In, Tl) monolayers. Solid State Commun..

[11] Zhang X., Liu Z., Hark S. (2007). Synthesis and optical characterization of single-crystalline AlN nanosheets. Solid State Commun..

[12] Borisenko D.P., Gusev A.S., Kargin N.I., Komissarov I.V., Kovalchuk N.G., Labunov V.A. (2019). Plasma assisted-MBE of GaN and AlN on graphene buffer layers. Jpn. J. Appl. Phys..

[13] Yang F., Jin L., Sun L., Ren X., Duan X., Cheng H., Xu Y., Zhang X., Lai Z., Chen W. (2018). Free-standing 2D hexagonal aluminum nitride dielectric crystals for high-performance organic field-effect transistors. Adv. Mater..

[14] Wang W.L., Zheng Y.L., Li X.C., Li Y., Zhao H., Huang L.G., Yang Z.C., Zhang X.N., Li G.Q. (2019). 2D AlN layers sandwiched between graphene and Si substrates. Adv. Mater..

[15] Chen Y.X., Liu K.L., Liu J.X., Lv T.R., Wei B., Zhang T., Zeng M.Q., Wang Z.C., Fu L. (2018). Growth of 2D GaN single crystals on liquid metals. J. Am. Chem. Soc..

[16] Wang W.L., Li Y., Zheng Y.L., Li X.C., Huang L.G., Li G.Q. (2019). Lattice structure and bandgap control of 2D GaN grown on graphene/Si heterostructure. Small.

[17] Zhang Z., Zhang S., Zhang L., Liu Z., Zhang H., Chen J., Zhou Q., Nie L., Dong Z., Pan G. (2021). Honeycomb-like gallium nitride prepared via dual-ion synergistic etching mechanism using amino acid as etchant. Chem. Phys. Lett..

[18] ElAfandy R.T., Majid M.A., Ng T.K., Zhao L., Cha D., Ooi B.S. (2014). Exfoliation of threading dislocation-free, single-crystalline, ultrathin gallium nitride nanomembranes. Adv. Funct. Mater..

[19] Syed N., Zhang Y., Zheng G., Wang L., Russo S.P., Esrafilzadeh D., McConville C.F., Kalantar-Zadeh K., Daeneke T. (2019). Wafer-Sized Ultrathin Gallium and indium nitride nanosheets through the ammonolysis of liquid metal derived oxides. J. Am. Chem. Soc..

[20] Wang X., Che S.-B., Ishitani Y., Yoshikawa A., Sasaki H., Shinagawa T., Yoshida S. (2007). Polarity inversion in high Mg-doped In-polar InN epitaxial layers. Appl. Phys. Lett..

[21] Pécz B., Nicotra G., Giannazzo F., Yakimova R., Koos A., Georgieva A.K. (2021). Indium Nitride at the 2D Limit. Adv. Mater..

[22] Singh A.K., Zhuang H.L., Hennig R.G. (2014). Ab initio synthesis of single-layer III-V materials. Phys. Rev. B.

[23] Jafari H., Ravan B.A., Faghihnasiri M. (2018). Mechanical and electronic properties of single-layer TiN and AlN under strain. Solid State Commun..

[24] Peng Q., Chen X.-J., Liu S., De S. (2013). Mechanical stabilities and properties of graphene-like aluminium nitride predicted from first-principles calculations. RSC Adv..

[25] Kourra M.H., Sadki K., Drissi L.B., Bousmina M. (2021). Mechanical response, thermal conductivity and phononic properties of group III-V 2D hexagonal compounds. Mater. Chem. Phys..

[26] Lv S.-J., Yin G.-X., Cui H.-L., Wang H.-Y. (2021). Electronic, vibrational, elastic, and piezoelectric properties of H-, F-f Functionalized AlN sheets. Phys. Status Solidi B.

[27] Tuoc V.N., Lien L.T.H., Huan T.D., Trung N.N. (2020). Structural, electronic and mechanical properties of few-layer GaN nanosheet: A first-principle study. Mater. Trans..

[28] Fabris G.S.L., Paskocimas C.A., Sambrano J.R., Paupitz R. (2021). One- and two-dimensional structures based on gallium nitride. J. Solid State Chem..

[29] Ahangari M.G., Fereidoon A., Mashhadzadeh A.H. (2017). Interlayer interaction and mechanical properties in multi-layer graphene, Boron-Nitride, Aluminum-Nitride and Gallium-Nitride graphene-like structure: A quantum-mechanical DFT study. Superlattices Microstruct..

[30] Peng Q., Sun X., Wang H., Yang Y., Wene X., Huang C., Liu S., De S. (2017). Theoretical prediction of a graphene-like structure of indium nitride: A promising excellent material for optoelectronics. Appl. Mater. Today.

[31] Peng Q., Liang C., Ji W., De S. (2012). A First Principles Investigation of the Mechanical Properties of g-TlN. Model. Numer. Simul. Mater. Sci..

[32] Luo Z.J., Yang Y.F., Yang X.Z., Lv B., Liu X.F. (2019). The mechanical properties and strain effect on the electronic properties of III-nitride monolayers: Ab-initio study. Mater. Res. Express.

[33] Faraji M., Bafekry A., Fadlallah M.M., Jappor H.R., Nguyen C.V., Ghergherehchi M. (2022). Two-dimensional XY monolayers (X = Al, Ga, In; Y = N, P, As) with a double layer hexagonal structure: A first-principles perspective. Appl. Surf. Sci..

[34] Rouhi S., Pourmirzaagha H., Bidgoli M.O. (2018). Molecular dynamics simulations of gallium nitride nanosheets under uniaxial and biaxial tensile loads. Int. J. Mod. Phys. B.

[35] Singh S., Raj B.M.R., Mali K.D., Watts G. (2021). Elastic Properties and nonlinear elasticity of the noncarbon hexagonal lattice nanomaterials based on the multiscale modelling. J. Eng. Mater. Technol..

[36] Le M.-Q. (2014). Atomistic Study on the tensile properties of hexagonal AlN, BN, GaN, InN and SiC sheets. J. Comput. Theor. Nanosci..

[37] Sarma J.V.N., Chowdhury R., Jayaganthan R. (2013). Mechanical behavior of gallium nitride nanosheets using molecular dynamics. Comput. Mater. Sci..

[38] Sakharova N.A., Antunes J.M., Pereira A.F.G., Fernandes J.V. (2017). Developments in the evaluation of elastic properties of carbon nanotubes and their heterojunctions by numerical simulation. AIMS Mater. Sci..

[39] Sakharova N.A., Antunes J.M., Pereira A.F.G., Chaparro B.M., Fernandes J.V. (2021). On the Determination of Elastic Properties of Single-Walled Boron Nitride Nanotubes by Numerical Simulation. Materials.

[40] Tapia A., Cab C., Hernández-Pérez A., Villanueva C., Peñuñuri F., Avilés F. (2017). The bond force constants and elastic properties of boron nitride nanosheets and nanoribbons using a hierarchical modeling approach. Physica E.

[41] Georgantzinos S.K., Kariotis K., Giannopoulos G.I., Anifantis N.K. (2020). Mechanical properties of hexagonal boron nitride monolayers: Finite element and analytical predictions. Proc. IMechE C J. Mech. Eng. Sci..

[42] Le M.-Q. (2015). Prediction of Young’s modulus of hexagonal monolayer sheets based on molecular mechanics. Int. J. Mech. Mater. Des..

[43] Giannopoulos G.I., Georgantzinos S.K. (2017). Tensile behavior of gallium nitride monolayer via nonlinear molecular mechanics. Eur. J. Mech. A Solids.

[44] Sakharova N.A., Pereira A.F.G., Antunes J.M., Chaparro B.M., Fernandes J.V. (2023). On the determination of elastic properties of indium nitride nanosheets and nanotubes by numerical simulation. Metals.

[45] Sakharova N.A., Antunes J.M., Pereira A.F.G., Chaparro B.M., Parreira T.G., Fernandes J.V. (2024). Numerical Evaluation of the Elastic Moduli of AlN and GaN Nanosheets. Materials.

[46] Li C., Chou T.W. (2003). A structural mechanics approach for the analysis of carbon nanotubes. Int. J. Solids Struct..

[47] Genoese A., Genoese A., Rizzi N.L., Salerno G. (2018). Force constants of BN, SiC, AlN and GaN sheets through discrete homogenization. Meccanica.

[48] Mayo S.L., Barry D., Olafson B.D., Goddard W.A. (1990). DREIDING: A generic force field for molecular simulations. J. Phys. Chem..

[49] Sakharova N.A., Pereira A.F.G., Antunes J.M. (2023). A Study of the mechanical behaviour of boron nitride nanosheets using numerical simulation. Nanomaterials.

[50] Zeng H., Zhi C., Zhang Z., Wei X., Wang X., Guo W., Bando Y., Golberg D. (2010). “White Graphenes”: Boron Nitride Nanoribbons via Boron Nitride Nanotube Unwrapping. Nano Lett..

[51] Nadeem A., Ali Raza M., Maqsood M.F., Ilyas M.T., Westwood A., Rehman Z.U. (2020). Characterization of boron nitride nanosheets synthesized by boron-ammonia reaction. Ceram. Int..

[52] Sakharova N.A., Pereira A.F.G., Antunes J.M., Chaparro B.M., Parreira T.G., Fernandes J.V. (2024). On the Determination of Elastic Properties of Single-Walled Nitride Nanotubes Using Numerical Simulation. Materials.

